# COCHLEA: Longitudinal Cognitive Performance of Older Adults with Hearing Loss and Cochlear Implants at 4.5-Year Follow-Up

**DOI:** 10.3390/brainsci14121279

**Published:** 2024-12-19

**Authors:** Julia Z. Sarant, Peter A. Busby, Adrian J. Schembri, Robert J. S. Briggs, Colin L. Masters, David C. Harris

**Affiliations:** 1 Department of Audiology and Speech Pathology, The University of Melbourne, 550 Swanston Street, Carlton, VIC 3010, Australia; 2 Cogstate Ltd., Level 2/161 Collins Street, Melbourne, VIC 3000, Australia; 3 The Royal Victorian Eye & Ear Hospital, 32 Gisborne Street, East Melbourne, VIC 3002, Australia; 4 The Florey Institute of Neuroscience and Mental Health, 30 Royal Parade, Parkville, VIC 3052, Australia

**Keywords:** hearing loss, dementia, cochlear implant, cognitive performance, cognitive decline, delay, risk reduction, executive function, working memory, older adults

## Abstract

Objectives: Hearing loss is highly prevalent in older adults and is independently associated with accelerated cognitive decline. Cochlear implants are usually the only effective treatment for people with severe–profound hearing loss, who have the highest risk of cognitive decline and dementia, however, very few receive them. Current evidence of the effects of cochlear implant use on cognitive decline/dementia outcomes is limited and unclear. This study aimed to investigate the effect of cochlear implant use on longitudinal cognitive performance, as this intervention may be an effective method of modifying cognitive outcomes for older adults with significant hearing loss. Methods: This prospective longitudinal observational study investigated cognitive performance in a convenience sample of older adults (mean age 74 years) with cochlear implants over 4.5 years post-implantation, comparing this with that of community-living adults with untreated hearing loss/normal hearing over 3 years (Australian Imaging, Biomarker and Lifestyle Flagship Study of Ageing; AIBL). All participants were assessed at 18-month intervals from baseline using the same measures. Panel regression was used to compare cognitive trajectories. Results: Cochlear implant users demonstrated significantly improved performance in executive function and working memory, as well as stability in attention, psychomotor function, and visual learning at 4.5-year follow-up. Comparatively, AIBL participants showed significantly greater worsening performance per year in attention and psychomotor function, and stability in working memory and visual learning at 3-year follow-up. Conclusions: Cochlear implant use may delay cognitive decline and/or improve cognitive performance in older adults with severe–profound hearing loss, providing proof-of-concept evidence of the positive effects of hearing intervention on cognitive performance in older adults with hearing loss.

## 1. Introduction

Hearing loss (HL) is highly prevalent in older adults [1], with 1.57 billion people affected worldwide. This number is projected to increase by 2050 to 2.45 billion due to population aging [2]. The evidence consistently supports an independent association between HL and accelerated cognitive decline/dementia in older adults, with HL estimated to be the equal largest worldwide potentially modifiable risk factor for dementia (7%) [3,4]. There is a positive correlation between degree of HL and dementia risk [5,6]. Older adults with severe–profound HL, for whom hearing aids provide little benefit, are thus at greatest risk of dementia [3,4]. Currently, 47.9 million people worldwide have severe–profound HL [7]. Many of this population, who often do not benefit from hearing aid use, disengage socially. Social isolation is a form of chronic stress associated with an increased release of stress hormones and a downward trajectory of psychological decline, poorer quality of life, and increased dementia risk [8,9].

Cochlear implants (CIs) are surgically implanted devices that electrically stimulate the auditory nerve directly, bypassing the defective auditory pathway [10]. They provide significantly improved perception of sound and communication for 90% of the treated population in terms of objective auditory benefit and effect size, reduced listening effort, and potentially reduced cognitive load [11,12,13]. With improved technology, communication and quality of life benefits are now substantial, and CIs are now indicated for adults with significant residual hearing in one or both ears, adults with unilateral hearing loss, adults with prelingual hearing loss, and even older adults in their ninth and tenth decades [11,14,15]. Although cochlear implantation can be life-changing for people with this degree of hearing disability, fewer than 10% of adults with severe–profound HL are implanted worldwide [16,17], and there is a lack of awareness of the comorbidities associated with HL [18]. Stronger health care provision mechanisms are needed to reduce the burden of untreated severe–profound HL [2].

The mechanisms of the association between HL and cognitive decline/dementia are unknown and likely multiple [19,20,21]. Hypothesized potentially modifiable mechanisms include the depletion and re-allocation of cognitive resources needed to process degraded auditory information; reduced auditory stimulation causing degenerative changes in brain structure and changes in function; and reduced environmental stimulation and social participation, leading also to degenerative changes in brain structure and function. These hypotheses are supported by evidence from neuroimaging studies showing associations between preserved hearing, a reduction in age-related medial lobe changes, and the preservation of auditory and other sensory cortical tissues irrespective of age [22]. Studies of individuals with HL show accelerated grey matter volume loss in the temporal lobe, which is directly involved in auditory processing [23,24], and also in the surrounding regions, such as the hippocampus, the para hippocampal gyrus, and the entorhinal cortex [24]. Poorer hearing is associated with degenerative changes in white matter microstructure [25], and evoked potential studies have shown visual and somatosensory cross-modal re-organization (the recruitment of auditory cortical regions by other sensory processes) dependent on HL severity [26,27]. Although it is unknown whether the association between HL and cognitive decline/dementia is causal, given this evidence, it is important to determine whether cochlear implantation could slow cognitive decline in older adults with severe–profound HL and thus improve function, social engagement, and quality of life in this population who often receive little benefit from hearing aid use.

The effects of hearing intervention on cognitive performance and dementia risk are unclear. There are no significant CI trials and only one randomized large-scale clinical trial (RCT) of hearing aids to date, with no non-intervention control group and a primary outcome of no benefit of device use on cognitive outcomes after 3 years, although a sub-group in the study at greater risk of cognitive decline showed a reduction in the rate of decline after hearing intervention [28]. No studies prior to 2015 prospectively investigated changes in cognitive performance after implantation with modern CIs [29]. Recent systematic reviews and meta-analyses have concluded that CIs may be beneficial but that the evidence is weak due to methodological limitations, and that well-designed studies with longer follow-up are needed [30,31]. These limitations include small sample sizes, e.g., [32,33]; short-term follow-up (only three studies beyond 2 years); assessment of cognition using only insensitive screening tools, e.g., [34,35]; auditory test administration, e.g., [35,36]; problematic or no statistical analyses and learning effects, e.g., [36,37,38]; modified, unverified test administration methods [30]; and a lack of control groups, e.g., [36,39,40]. Given the long prodromal period for dementia, long-term RCTs are impractical for studying dementia, and larger-scale observational studies of cognitive performance and dementia outcomes after treatment of HL are needed [30,31].

The current study (COCHLEA: Cochlear Implant Outcomes and Cognitive Health—Longitudinal Evaluation of Adults) investigated the effect of CI use on cognitive outcomes over 4.5 years in implanted older adults with severe–profound HL, compared with outcomes of community-living older adults with either untreated HL or normal hearing (a PTA of less than 20 dB hearing level (HL); World Health Organization [41]) who were participants of the Australian Imaging, Biomarker and Lifestyle Flagship Study of Ageing (AIBL) [42]. It was hypothesized that improved hearing would reduce cognitive load and improve cognitive performance in the group of older adults with significant hearing disability who received CIs. The effects of various participant characteristics and amounts of device use on cognitive performance were explored. This study addresses many of the methodological limitations of previous studies.

## 2. Materials and Methods

### 2.1. Participants

Data were collected at baseline prior to cochlear implantation from a convenience sample of 101 adults aged ≥ 60 years with severe–profound HL who were eligible for and had chosen to receive a CI prior to recruitment into this study, had no diagnosed or suspected dementia, had passed the Mini Mental Screening test (MMSE [43]), had no language difficulties that prevented them from completing the assessment protocol, and who chose to participate. Follow-up data were collected from a subset of the first participants who reached any or all follow-up points (18, 36, and 54 months) by the end of May 2023. Participants were patients of either the Royal Victorian Eye and Ear Hospital Cochlear Implant Clinic (Australia) or the Northern Cochlear Implant Programme (New Zealand). As this study is ongoing, most participants who had not reached these follow-up points remain in the study and will be assessed in future. Thirty-three CI participants were lost to follow-up. Reasons for withdrawal included death (N = 5), dementia diagnosis (Alzheimer’s; N = 1), poor health (e.g., chronic illness, mobility problems, palliative care; N = 7), non-use of the device due to poor outcomes (N = 7), finding completion of the assessment battery and travel for assessment too onerous (N = 7), spousal carer responsibilities (N = 1), and lack of reply to contact for follow-up for two or more reviews (n = 5).

Outcomes for the CI group were compared up to 3 years with outcomes for 100 participants aged ≥60 years with untreated HL or normal hearing recruited from a large longitudinal cohort study of community-living older adults (Australian Imaging, Biomarker and Lifestyle Flagship Study of Ageing; AIBL) [42]. The AIBL study was launched in 2006 and is a large Australian prospective cohort study investigating the natural history of Alzheimer’s disease from preclinical onset through to the development of dementia. The study databank includes brain imaging, biospecimens, and clinical and cognitive performance data, which are being used to investigate which cognitive characteristics, biomarkers, and health and lifestyle factors are predictive of the development and progression of Alzheimer’s disease. The AIBL sample included older adults both without HL and with HL who did not use HAs, as would be expected in a representative sample of the general population. Assessments were conducted at the same time intervals and using the same assessment battery as for CI participants with subsets of the first AIBL participants to reach the 18- and 36-month follow-up points. The AIBL follow-up sample size at 54 months was too small to enable meaningful analysis. Demographic and audiological data for all participants are summarized in Table 1.

### 2.2. Measures

#### 2.2.1. Demographic Characteristics

Demographic characteristics included chronological age, sex, degree of HL, years of education, presence of medical conditions, anxiety, depression, falls, smoking, physical activity, living arrangements, living alone, and retirement status (Table 1).

#### 2.2.2. Cognitive Performance and Dementia Diagnosis

Dementia screening was conducted at baseline using The Mini Mental State Examination (MMSE; [43]). As per test instructions, a cut-off score of 24 was used to identify people with cognitive impairment. Cognition was thereafter assessed using the computerized Cogstate Brief Battery (CSBB) and the Cogstate Groton Maze Learning Test (GMLT) only due to their minimal practice effects, high sensitivity to small changes in cognitive performance and longitudinal decline in older adults, high reliability and specificity for different cognitive functions, and visual (non-auditory) presentation, which makes these tools highly suitable for use with people with HL [44,45,46]. The full battery (CSBB and GMLT) is relatively quick to administer (30 min). Test-retest reliability for each subtest ranges from 0.84 to 0.94 [44].

The CSBB subtests include psychomotor function (Detection test), attention (Identification test), working memory (One Back Test), and visual learning (One Card Learning). The GMLT assesses executive function and takes the longest time to complete. For this reason, GMLT data were not available for the AIBL comparison group.

All cognitive assessments were administered by a small team of audiologists trained and supervised by a neuropsychologist. Wherever possible, the same audiologist would see a particular participant to ensure continuity of the relationship and stability of the assessment conditions. Risk of bias regarding the results was eliminated, as after the practice/training session, participants completed the assessment in a quiet room alone (either at the university or at home), with de-identified results automatically uploaded to the centralized Cogstate platform for automated scoring, in which process response speed and accuracy were transformed to yield normalized data distributions [44,45]. As the participants in this study were older adults and some did not regularly use a computer, particular care was taken during training to ensure participants were able to adequately use the mouse to complete the required tasks before data collection was initiated. Care was also taken to ensure that visual function was adequate to facilitate reading the computer screen to understand the instructions and perform the cognitive tasks. Tasks were administered in the following order as determined by the Cogstate software (version 7.0): GMLT (7 min), Detection (3 min), Identification (3 min), One Card Learning (6 min), and OneBack (4 min). Written instructions were presented on the computer screen prior to each task. Each participant worked through the assessment battery once for training and a second time (after a break) for data collection. During the practice/training session, audiologists provided assistance with understanding the instructions if needed. The time taken to complete the assessment battery varied between participants depending on reaction times and accuracy, but averaged 30 min.

For all tasks, both speed and accuracy of responses were recorded, with a single performance measure selected on the basis that it was derived from a normal data distribution, had no ceiling or floor effects, had an unrestricted range, and good stability, sensitivity, and reliability to change [47,48]. For samples that are not entirely cognitively impaired, the Cogstate protocol specifies that working memory (OneBack), psychomotor function (Detection), and visual attention (Identification) are scored based on speed or reaction time (milliseconds); thus, lower scores indicate better cognitive performance in these tasks. Visual learning (One Card Learning) is scored according to accuracy (the proportion of correct answers), and is thus reverse-scored, with higher scores indicating better cognitive performance. Primary outcome (raw) scores and not z-scores were used in all statistical analyses to enable the examination of the relationship between age and cognitive performance, as z-scores are standardized for age. The CSBB and GMLT are designed for profiling cognitive performance and change over time in people both with and without dementia, but not for diagnostic use.

Dementia outcomes were determined via medical history completed at each timepoint, in which participants and their families were asked to report any medical diagnoses of cognitive impairment (mild cognitive impairment or dementia). Screening for dementia was not conducted after participants enrolled in this study.

#### 2.2.3. Audiological Assessment

HL was assessed by an audiologist from the research team in a sound-proof booth at baseline for all participants, and at each subsequent follow-up in a booth or quiet room (for participants who were assessed at home) with background noise measured at 40–50 dB(A) or less, using gold-standard audiometric practice—pure tone audiometry [49]. It should be noted that participants with severe–profound hearing loss would not hear background noise below approximately 60 db(A). Audiological assessment included air and bone conduction hearing thresholds, speech discrimination, and tympanometry. Four-frequency pure tone averages (PTAs; average of four hearing thresholds at 500, 1000, 2000 and 4000 kHz) were calculated, with a PTA of greater than 20 dB hearing level (HL) identifying HL, as per the World Health Organization criteria [41]. As CI indications have broadened from profound bilateral deafness, with limited use of amplification prior to implantation, to significant residual hearing and speech perception ability in one or both ears, and HL also usually declines over a long period, it has become difficult to accurately measure the duration and extent of auditory deprivation in CI candidates prior to surgery [50]. For this reason, information about the duration of HL prior to implantation is not provided.

#### 2.2.4. Speech Perception Benefit

Speech perception assessments were conducted at baseline (prior to implantation) and at 18-month follow-ups with all participants wearing their usual devices (if any) to ensure the best possible (and usual) aided listening condition. Post-implantation, 49 (90.7%), 27 (73%), and 14 (56%) of participants assessed at 18, 36, and 54 months chose to wear a hearing aid in their non-implanted ear (i.e., were bimodal device users). One, seven, and six CI participants were implanted bilaterally at the 18-, 36-, and 54-month follow-ups, respectively. Speech perception assessment included recorded consonant–vowel–consonant (CVC) monosyllabic words (50-word lists; scored for words and phonemes correct) presented at 65dBSPL unaided and in quiet in the left ear, right ear, and binaurally at baseline and in the preferred aided condition for participants post-CI (CI alone, bilateral CIs, CI plus hearing aid). Speech Reception Threshold (SRT) testing was conducted using Bamford–Kowal–Bench-like sentence lists presented at 65dBSPL in 4-talker babble background noise, with an adaptive noise level dependent on sentence scores. The final measure reflected the signal-to-noise ratio at which 50% of the key words were correct. Speech and background noise were presented 1 m in front of participants via a single speaker in free field. The non-test ear was masked in the unilateral listening conditions using white noise set 30 dBHL above the average of the participant’s 1000 and 2000 kHz thresholds, or at 60 dBHL for participants with severe–profound loss pre-operatively. The mean words correct score for each sentence was used to calculate speech-in-noise perception results for the right ear, left ear, and binaurally.

#### 2.2.5. Genetic Screening

Saliva samples were taken at baseline from all participants to identify carriers of the Apolipoprotein (APOE) ε4 allele, the strongest known genetic risk factor for late-onset Alzheimer’s dementia [51,52], as carrier status could influence cognitive outcomes. DNA genotyping and reporting was conducted by New South Wales Pathology.

#### 2.2.6. Medical Health History

An extensive medical health history was taken at baseline and repeated at each follow-up. This included a personal health history documenting the presence of cardiovascular disease, diabetes, falls, smoking, illicit drug use, medication use, and family history of neurological illness and mental health disorders. Participants were classified as having cardiovascular disease if they reported a diagnosis of one or more of stroke, myocardial infarction, angina, or hypertension.

#### 2.2.7. Mood

Self-reported anxiety and depression were measured using the Hospital Anxiety and Depression Scale [53], with participants choosing to answer this questionnaire either at the time of their assessment or at home afterwards. This tool is widely used, with reported specificities and sensitivities for anxiety and depression, respectively, of 0.78/0.9 and 0.79/0.83.

#### 2.2.8. Living Arrangements/Living Alone/Social Isolation

Living alone status was recorded at all assessment points, as living alone is a recognized risk factor for cognitive decline [54,55]. Living arrangements were also recorded (i.e., living at home, with relatives, independently in a retirement village, in residential aged care). Self-reported social isolation was also measured using the Lubben Social Network Scale (LSNS; [56]). This is a brief questionnaire about perceived support from family and friends designed to assess social isolation in older adults. The questionnaire comprises an equally weighted number of items measuring size, frequency, and closeness of the respondent’s social network. This information was not available for AIBL participants.

#### 2.2.9. Health-Related Quality of Life

Health-related quality of life for CI participants was measured at all assessment points using the Health Utilities Index-3 Quality of Life Questionnaire (HUI-3) hearing disability scale [57]. This information was not available for AIBL participants.

### 2.3. Statistical Analysis

#### 2.3.1. Cognitive Trajectories of the CI and AIBL Groups

The trajectory of cognition over time was analysed for the CI and AIBL groups separately using panel regression equations of the form:

(1) $$\begin{array}{ll} Y_{i,t} & =\mu_{0}+\delta_{1}D_{1,t}+\delta_{2}D_{2,t}+\delta_{3}D_{3,t}\\ & \;\;+\psi_{1}{\mathrm{Age}70}_{i}+\psi_{2}{\mathrm{Female}}_{i}+\psi_{3}{\mathrm{HigherEd}}_{i}+U_{i,t}, \end{array}$$

where $Y_{i,t}$ denotes the cognitive outcome for participant i at time t=0,1,2,3 (corresponding to baseline, 18-month, 36-month, and 54-month time periods), $D_{1,t}$, $D_{2,t}$ and $D_{3,t}$ are time period indicators, ${\mathrm{Age}70}_{i}$ is age at baseline relative to 70 years, and ${\mathrm{Female}}_{i}$ and ${\mathrm{HigherEd}}_{i}$ are indicators of female sex and education attainment beyond 12 years, respectively. The analysis of the AIBL group omitted the 54-month term since observations were not available for that period. The coefficient $\mu_{0}$ represents the mean cognitive outcome at baseline for 70-year-old males with no more than 12 years of education. The coefficients $\delta_{1}$, $\delta_{2}$, and $\delta_{3}$ measure the changes in mean cognitive score from baseline to 18, 36, and 54 months, respectively, controlling for age, sex, and education. Estimation allowed for an unbalanced panel with heteroskedasticity-consistent standard errors clustered at the participant level to allow for intra-participant observation dependence. The individual significance of each of the $\delta_{1}$, $\delta_{2}$, and $\delta_{3}$ coefficients was evaluated using a Holm–Bonferroni adjustment to control the overall level for the trajectory tests in each equation at 0.05.

Comparative analyses of the cognitive trajectories of bimodal versus unilateral CI users were not performed due to small sample sizes.

#### 2.3.2. Device Use and Cognitive Trajectories

An additional analysis was carried out for the CI group to investigate whether the cognitive trajectories in Equation (1) varied with CI usage. Equation (1) was extended to

(2) $$\begin{array}{ll} Y_{i,t} & =\mu_{0}+\delta_{1}D_{1,t}+\delta_{2}D_{2,t}+\delta_{3}D_{3,t}\\ & \;\;+\kappa_{1}\left(D_{1,t}\times {\mathrm{HighUse}}_{i,t}\right)+\kappa_{2}\left(D_{2,t}\times {\mathrm{HighUse}}_{i,t}\right)+\kappa_{3}\left(D_{3,t}\times {\mathrm{HighUse}}_{i,t}\right)\\ & \;\;+\psi_{1}{\mathrm{Age}70}_{i}+\psi_{2}{\mathrm{Female}}_{i}+\psi_{3}{\mathrm{HigherEd}}_{i}+U_{i,t}, \end{array}$$

with ${\mathrm{HighUse}}_{i,t}$, an indicator for average daily CI use exceeding 14 h, where average daily CI use at time t was computed as the cumulative weighted average over all periods up to and including time t. The coefficients $\kappa_{1}$, $\kappa_{2}$, and $\kappa_{3}$ denote the additional changes, relative to $\delta_{1},\delta_{2}$, and $\delta_{3}$, in mean cognition score at each follow-up due to high usage of the CI. The possibility of a CI usage effect at each time period was evaluated by the individual significance of each of the $\kappa_{1}$, $\kappa_{2}$, and $\kappa_{3}$ coefficients, with a Holm–Bonferroni adjustment to control the overall level of the CI usage test at 0.05.

#### 2.3.3. Comparative Cognitive Performance: CI Versus AIBL Groups

The trajectories of cognition for the CI and AIBL groups were compared using a panel regression equation of the form

(3) $$\begin{array}{ll} Y_{i,t} & =\alpha_{i}+\delta_{1}D_{1,t}+\delta_{2}D_{2,t}\\ & \;\;+\lambda_{1}\left(D_{1,t}\times {\mathrm{CI}}_{i}\right)+\lambda_{2}\left(D_{2,t}\times {\mathrm{CI}}_{i}\right)+U_{i,t} \end{array}$$

where $\delta_{1}$ and $\delta_{2}$ are the changes in mean cognitive scores for the AIBL group at each follow-up, and $\lambda_{1}$ and $\lambda_{2}$ are the differences between the CI and AIBL changes in mean cognitive scores. The difference between the CI and AIBL trajectories was evaluated through the individual significance of $\lambda_{1}$ and $\lambda_{2}$, with a Holm–Bonferroni adjustment to control the overall level of the test at 0.05. The term $\alpha_{i}$ represents participant-specific time-invariant fixed effects to control for all individual baseline characteristics, included to control for any differences, observed or unobserved, between the CI and AIBL groups.

#### 2.3.4. Comparative Cognitive Performance: CI vs. AIBL Group with HL Only

Although a primary aim of this study was to compare cognitive performance outcomes for CI participants to those of a representative group of older adults in the community, a secondary analysis was performed using a comparison group of AIBL participants reduced to only those with HL. The statistical methods used were identical to those for the comparison of the CI vs. (whole) AIBL group.

#### 2.3.5. Sensitivity Analysis of Differences in Education Between the CI and AIBL Groups

The proportion of CI participants with higher education was significantly lower than that for the AIBL group. Since education may plausibly affect cognitive outcomes, a sensitivity analysis of the results from Equation (3) was carried out by extending the specification to

(4) $$\begin{array}{ll} Y_{i,t} & =\alpha_{i}+\delta_{1}D_{18,t}+\delta_{2}D_{36,t}\\ & \;\;+\lambda_{1}\left(D_{18,t}\times {\mathrm{CI}}_{i}\right)+\lambda_{2}\left(D_{36,t}\times {\mathrm{CI}}_{i}\right)\\ & \;\;+\eta_{1}\left(D_{18,t}\times {\mathrm{AIBL}}_{i}\times {\mathrm{HigherEd}}_{i}\right)+\eta_{2}\left(D_{36,t}\times {\mathrm{AIBL}}_{i}\times {\mathrm{HigherEd}}_{i}\right)\\ & \;\;+\eta_{3}\left(D_{18,t}\times {\mathrm{CI}}_{i}\times {\mathrm{HigherEd}}_{i}\right)+\eta_{4}\left(D_{36,t}\times {\mathrm{CI}}_{i}\times {\mathrm{HigherEd}}_{i}\right)+U_{i,t} \end{array}$$

The possible variation in cognition trajectories with education was evaluated through the individual significance of $\eta_{1},\eta_{2},\eta_{3}$, and $\eta_{4}$, with a Holm–Bonferroni adjustment to control the overall level of the test at 0.05.

#### 2.3.6. Relations Between Baseline and Follow-Up Cognitive Performance

Four studies to date have reported greater cognitive improvements for CI users who performed more poorly pre-operatively [38,39,58,59]. However, the methods used to arrive at this determination in these studies could not exclude statistical regression to the mean, as opposed to the hearing intervention having genuinely differing effects at different cognition levels. These methods included the use of Pearson and Spearman rank order correlations, which will confound regression to the mean with any real effect if one existed and counting the number of tests of different cognitive domains on which performance improved. Only one study included a non-intervention control group [58], which unfortunately comprised only people with normal hearing, who were therefore not at the same risk of cognitive decline as those with HL. Thus, the outcomes of poor performers in the non-treatment group could not be examined to differentiate any real effect from regression to the mean. To meaningfully analyse cognitive improvement and baseline cognition, and the association between them, it was necessary to separately model both any real effects and regression-to-the-mean effects. This was achieved with the testing equation

(5) $$\begin{array}{ll} Y_{i,t}^{*}-Y_{i,0}^{*} & =\left(\rho_{1}-1\right)\left(Y_{i,0}^{*}\times D_{1,t}\right)+\left(\rho_{2}-1\right)\left(Y_{i,0}^{*}\times D_{2,t}\right)\\ & \;\;+\phi_{1}\left({\mathrm{CI}}_{i}\times Y_{i,0}^{*}\times D_{1,t}\right)+\phi_{2}\left({\mathrm{CI}}_{i}\times Y_{i,0}^{*}\times D_{2,t}\right)\\ & \;\;+\delta_{1}^{*}D_{1,t}+\delta_{2}^{*}D_{2,t}+\lambda_{1}^{*}\left({\mathrm{CI}}_{i}\times D_{1,t}\right)+\lambda_{2}^{*}\left({\mathrm{CI}}_{i}\times D_{2,t}\right)\\ & \;\;+\psi_{1,1}^{*}\left({\mathrm{Age}70}_{i}\times D_{1,t}\right)+\psi_{1,2}^{*}\left({\mathrm{Female}}_{i}\times D_{1,t}\right)+\psi_{1,3}^{*}\left({\mathrm{HigherEd}}_{i}\times D_{1,t}\right)\\ & \;\;+\psi_{2,1}^{*}\left({\mathrm{Age}70}_{i}\times D_{2,t}\right)+\psi_{2,2}^{*}\left({\mathrm{Female}}_{i}\times D_{2,t}\right)+\psi_{2,3}^{*}\left({\mathrm{HigherEd}}_{i}\times D_{2,t}\right)\\ & \;\;+V_{i,t}, \end{array}$$

where $Y_{i,t}^{*}=Y_{i,t}/\sigma_{t}$, $\sigma_{t}=\mathrm{sd}\left(Y_{i,t}\right)$, and $\rho_{t}=\mathrm{cor}\left(Y_{i,t},Y_{i,0}\right)$ for t=1,2. Regression to the mean at each follow-up time period is captured by the first two terms in this equation, with the second two terms capturing any further relationship between cognitive trajectories and baseline cognition attributable to CI use. If estimates of $\phi_{1}$ and/or $\phi_{2}$ are significant, then this may be evidence that the CI treatment effect is correlated with baseline cognition. The significance of these two coefficients was evaluated in each equation with a Holm–Bonferroni adjustment to control the overall level at 0.05. Derivations of the testing equations are given in Appendix A.

## 3. Results

### 3.1. Participant Characteristics

Table 1 shows the demographic and HL data for all participants and the numbers of participants assessed at each follow-up. One hundred and one CI participants (41.6% female; mean [range] age 73 [61–90] years old) were implanted with either the CI512, CI522, CI532, CI6122 or CI632 Cochlear Nucleus implants (contour array, slim, straight, or slim contour). All participants except one (who used SPEAK [60]) used the ACE speech processing strategy [61] with either the Cochlear^TM^ Nucleus^®^ 6, 7, Kanso^®^ (CP950), or Kanso^®^ 2 (CP1150) speech processor (Sydney, Australia). A small number of CI participants were implanted bilaterally: 0 at baseline, 1 at 18 months (1.9%), 7 at 36 months (18.9%), and 6 at 54 months (24%). At baseline, all but one CI participant (who received no benefit) used a hearing aid in at least one ear, although for four participants usage was low due to minimal benefit. At 18 months, 49 (90.7%) of the follow-up sample (n = 54) were bimodal (i.e., also used a hearing aid in their non-implanted ear); at 36 months, 27 (73%) of the follow-up sample (n = 37), were bimodal; and at 54 months, 14 (56%) of the follow-up sample (n = 25) were bimodal. One hundred AIBL participants (55% females; mean [range] age 74 [67–85] years old) with either untreated HL (mean pure tone average of 21 dBHL) or normal hearing (47%) did not use any devices.

There were no significant differences between the participant groups at baseline for age, sex, falls, depression, APOε4 allele carrier status, smoking, living alone, or retired status. Only one CI participant and no AIBL participants lived in residential aged care at baseline only, and not in the general community. At baseline, CI participants had greater HL, were less likely to be tertiary-educated, and more likely to have diabetes and anxiety. They were also more likely to have cardiovascular disease both at baseline and at 18-month follow-up. Baseline differences between the groups were controlled using participant-specific fixed effects (see statistical analysis section). The cardiovascular difference was not controlled in the analyses, as if this affected cognitive performance it could be expected to worsen the CI group’s results, not improve them. Follow-up data on HL are missing for 11 and 1 AIBL participants at the 18-month and 36-month follow-ups due to the inability to conduct in-person audiometry during COVID-19 outbreaks. Education information was not given by two CI participants.

### 3.2. CI Usage and Speech Perception Benefits

Table 2 shows objective device usage (obtained using data logging) and speech perception benefits obtained in both quiet and noisy listening conditions. Despite almost 2 years of COVID-19 lockdowns and associated social isolation in Melbourne, Australia, and in New Zealand, the CI participants demonstrated excellent device mean usage of over 12 h per day at the 18-, 36-, and 54-month follow-ups. Mean CVC Word scores in quiet listening conditions improved significantly (*p* = 0.000) at the 18-month follow-up, with a non-significant trend of ongoing improvement from a baseline score of 42.8% to a mean score of 78.1% at the 54-month follow-up. SRT scores in noise also improved significantly from baseline to the 18-month follow-up (*p* = 0.000), with the required signal-to-noise ratio decreasing from 10.94 to 3.66 at the 54-month follow-up. Although the mean required ratio showed a reducing ongoing trend through to the 54-month follow-up, the changes were not statistically significant between each follow-up point.

### 3.3. Cognitive Performance

#### 3.3.1. Baseline Cognitive Performance

Table 3 shows the baseline mean raw scores for CI participants only on the GMLT of executive function (this was not conducted as part of the AIBL study) and the mean baseline and follow-up raw scores on the CSBB subtests for both participant groups. At baseline, the mean cognitive scores for the AIBL participants were significantly better than for the CI participants across all CSBB subtests. MMSE baseline screening of mean group performance for the AIBL participants was also significantly better than for the CI participants.

#### 3.3.2. Cognitive Trajectories of the CI and AIBL Groups

Table 4 shows cognitive performance at follow-up for the CI participants. Joint tests of absolute mean raw cognitive performance scores on all subtests for the CI participant group at baseline versus all follow-ups through to 54 months were conducted, controlling for differences in age, sex, and education, and applying Holm–Bonferroni corrections for multiple comparisons. Table 4 provides estimates of Equation (1) for cognitive performance at follow-up for the CI participants. The estimated baseline mean executive function (GMLT) score for 70-year-old male participants without higher education is ${\hat{\mu }}_{0}=56.381.$ The mean GMLT score changed from baseline by ${\hat{\delta }}_{1}=-9.825$, ${\hat{\delta }}_{2}=-14.673$, and ${\hat{\delta }}_{3}=-17.579$ at 18 months, 36 months, and 54 months, respectively, with the latter two changes significant at the 5% level following Holm–Bonferroni correction. These lower GMLT scores represent improvements in executive function. Similarly, for working memory (ONB), the significant 54-month coefficient ${\hat{\delta }}_{3}=-0.050$ demonstrated an improvement in working memory relative to the baseline mean of ${\hat{\mu }}_{0}=2.962.$ There was no evidence of significant change in visual attention (IDN), visual learning (OCL), or psychomotor function (DET) performance for CI participants across the follow-up period.

Table 5 provides the same results for the whole group of AIBL participants from baseline through to 36 months. In the first panel, for the test of psychomotor function (DET), the baseline mean of ${\hat{\mu }}_{0}=2.535$ was found to increase significantly by ${\hat{\delta }}_{1}=0.039$ at 18 months and ${\hat{\delta }}_{2}=0.099$ at 36 months. These higher DET scores represent deterioration in psychomotor function. Similar results for the identification test of visual attention (IDN) also demonstrate significant deterioration at both 18 and 36 months. AIBL mean raw scores did not change significantly for working memory (ONB) or visual learning (OCL).

The second panel of Table 5 provides results only for AIBL participants who exhibited HL during the study (N = 56). The only change in results for this versus the previous analysis was for visual attention (IDN). Although the point estimates indicated towards decline, with a *p*-value of 0.036 at the 18-month follow-up, this result was not significant after application of the Bonferroni correction. This is likely due to an effect of reduced sample size, as excluding participants with normal hearing is unlikely to improve cognitive performance outcomes due to HL status.

The control variables of age, sex, and education are also reported in Table 4 and Table 5. Older CI group participants were more likely to score more poorly on average on all subtests, while older AIBL participants were more likely to score more poorly on all subtests except visual learning (OCL). There were no differences in performance due to sex or education within either group.

#### 3.3.3. Device Use and Cognitive Trajectories

Panel regression modelling, controlling for age, sex, and education, investigated whether there was a dose–response relationship between device usage and rate of cognitive improvement for CI users (Table 6). Although the mean daily usage time at all follow-up timepoints was high at over 12 h, participants who used their devices ≥14 h per day (most waking hours) showed a significantly greater magnitude of improvement in cognitive performance at the 18-month follow-up on all subtests except visual learning (OCL), compared with those who used their devices less, for whom significant improvement from baseline was not seen until the 36-month follow-up.

**Figure 1:**
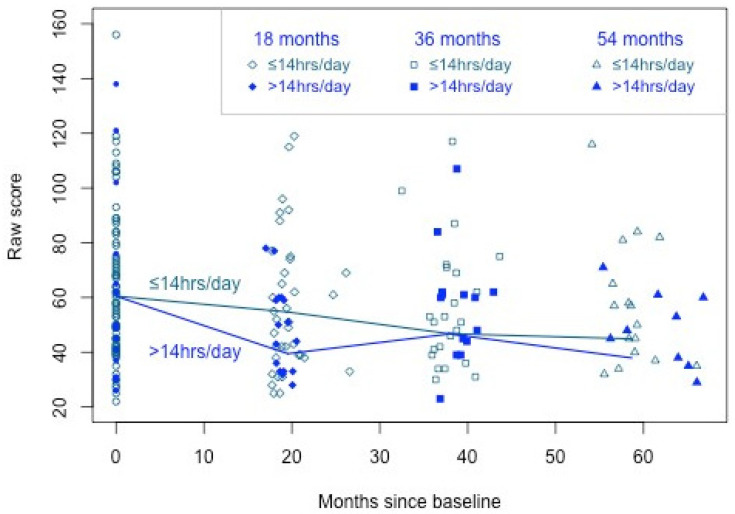
Longitudinal executive function cognitive performance trajectories for higher (≥14 h per day) and lower CI users from 18- to 54-month follow-up.

Table 6 shows estimates of Equation (2) for the CI participants classified by CI usage. Focussing on the 18-month results for executive function (GMLT), the mean change from baseline for low-use participants was estimated to be ${\hat{\delta }}_{1}=-5.138$, and the additional change from baseline for high-use participants was ${\hat{\kappa }}_{1}=-19.560$, significant at the 5% level following Holm–Bonferroni correction. While the mean GMLT score across all participants did not significantly improve by 18 months, these results imply the mean GMLT score did improve by 18 months for participants with high CI usage. The differences in GMLT between high and low CI usage at 36 and 54 months were not significant. These results are illustrated in Figure 1. The significant improvement at 18 months for those with high usage (>14 h per day) is evident from the steeper decline than for those with lower usage (≤14 h per day). The overall improvement for all participants by 36 and 54 months is evident from the decline in both means by these time points.

**Figure 2:**
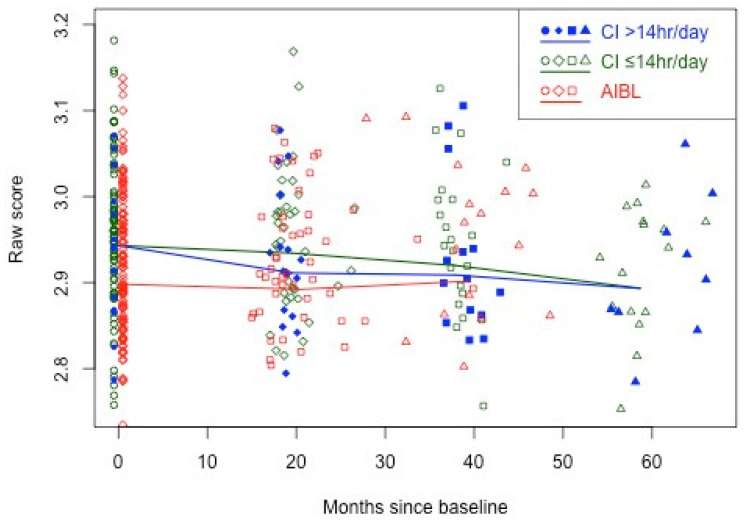
Longitudinal working memory cognitive performance trajectories for higher (≥14 h per day) and lower CI users from 18- to 54-month follow-up, plus AIBL group performance through to 36-month follow-up for comparison.

The same analysis also demonstrates significant improvements for high-CI-use participants in working memory, psychomotor function, and visual attention at 18 months. Figure 2 illustrates the results for working memory (ONB), with AIBL observations also included. The figure shows the overall decrease (improvement) in ONB scores across all CI participants, with more rapid initial improvement to 18 months for the high-CI-usage participants. For comparison, Figure 2 also shows the overall stability in ONB scores for the AIBL participants.

#### 3.3.4. Comparative Cognitive Performance: CI Versus AIBL Groups

Comparison of the cognitive performances of the CI and AIBL groups on the CSBB was performed using panel multiple regressions of the form of Equation (3), including individual fixed effects $\alpha_{i}$ to allow for all time-invariant group differences at baseline. The results are presented in the first panel of Table 7.

Considering visual attention (IDN), the estimates of ${\hat{\delta }}_{1}=0.039$ and ${\hat{\delta }}_{2}=0.067$ are the mean changes from baseline for the AIBL participants, controlling for the individual fixed effects. The estimates $\lambda_{1}=-0.028$ and $\lambda_{2}=-0.060$ quantify the differences of the CI group means from the AIBL group means at 18 months and 36 months. Both are negative and significant following Holm–Bonferroni adjustment, indicating superior changes in IDN outcomes for the CI group relative to the AIBL group. Similar analysis also demonstrates a superior trajectory in psychomotor function (DET) outcomes for the CI group at 36 months. There were no significant differences in performance between the groups for working memory (ONB) or visual learning (OCL).

Although a main aim of this study was to compare the cognitive performance of CI participants with a sample of older, community-living adults representative of the general population (including people with and without HL, as would be expected in the community), panel 2 of Table 7 also shows the results of a further panel multiple regression analysis comparing cognitive performance outcomes for treated versus untreated groups with hearing loss only. In this analysis, although superior changes were still seen in psychomotor function (DET) outcomes for the CI group at 36 months, there were no significant differences in visual attention (IDN) performance at either timepoint. Again, there were no significant differences in performance between the groups for working memory (ONB) or visual learning (OCL).

#### 3.3.5. Sensitivity Analyses for Differences in Education Between the CI and AIBL Groups

As the proportion of the CI group with higher education was significantly lower than that for the AIBL group, a sensitivity analysis was conducted to control for any effects of this difference on the trajectory of cognitive change for both groups (Table 8). After Holm–Bonferroni corrections were applied, only one interaction was significant: AIBL participants with higher education performed significantly better in psychomotor function than those without higher education at 36 months. There was no effect of education on the overall outcomes reported in Section 3.3.4.

The same analysis was repeated for the CI group versus only AIBL participants with HL (Table 8), yielding the same results.

#### 3.3.6. Relations Between Baseline and Follow-Up Cognitive Performance

As discussed previously, four studies to date have reported greater cognitive improvements for CI users who performed more poorly pre-operatively [38,39,58,59]. However, the methods used to arrive at this conclusion in these studies could not exclude regression to the mean. Table 9 (CI and AIBL trajectories versus baseline) shows the results of separate modelling for both real effects and regression-to-the-mean effects. Estimates of $\phi_{1}$ and $\phi_{2}$ (baseline to 18-month CI; baseline to 36-month CI; see equation in Section 2.3.5;) were not significant; thus, there was no evidence that CI treatment effects varied according to baseline cognitive performance. Further, the table shows negative estimates for $\rho_{1}$ and $\rho_{2}$ for all subtests (baseline: 18 months; baseline: 36 months), significant after Holm–Bonferroni corrections for working memory (ONB) and visual attention (IDN), indicating regression-to-the-mean effects only.

#### 3.3.7. The Effect of Attrition on Cognitive Performance Outcomes

There were 54 withdrawals of CI participants from this study, 40 of which occurred at baseline when participants found the combination of cognitive assessments and questionnaires too onerous to complete. Generally, it is plausible that a pattern of withdrawals systematically associated with cognitive abilities may bias the cognitive trajectory outcomes, which could then be artificially inflated. Appendix B presents a sensitivity analysis to investigate this possibility, excluding all 40 participants who withdrew at baseline and including a table showing reasons for withdrawal and the numbers of these at each timepoint. When compared with the results in Table 4, which includes the whole sample, the results are almost unchanged, and are in fact strengthened, with two additional significant findings of improvement for CI participants (at 18 months for executive performance and at 36 months for working memory).

#### 3.3.8. Dementia Outcomes

At 54 months, one CI group participant had been diagnosed with Alzheimer’s dementia, and another had neurological changes post-stroke, but no dementia diagnosis. In the AIBL group at the 36-month follow-up, 15 participants had been diagnosed with mild cognitive impairment and 1 had been diagnosed with dementia related to Parkinson’s disease.

## 4. Discussion

Despite significantly poorer baseline cognitive performance across the assessment battery and many more risk factors for cognitive decline (poorer education, anxiety, diabetes, cardiovascular disease, significantly poorer hearing, and a greater effect of age on cognitive performance), the CI group demonstrated no decline in any measures from baseline and significantly improved performance in executive function and working memory from 36- and 54-month follow-up, respectively. The AIBL group, with significantly less HL and fewer cognitive decline risk factors, demonstrated comparatively greater worsening cognitive performance in two of the four subtests of the Cogstate Battery. This study provides proof-of-concept evidence of the effects of cochlear implantation on cognitive function in older adults. The cognitive improvement/stability in the CI group is likely mediated by neural plasticity after peripheral stimulation with a CI. Animal studies have shown changes in auditory cortex neural response properties and functional organization after cochlear implantation [62,63], while human electrophysiological studies have demonstrated a reversal of the cortical re-organization of the auditory cortex for visual processing coincident with improved speech perception and cognitive performance [64]. Imaging studies have shown the restoration of metabolic activity in the primary auditory cortex to almost normal levels after CI use [65].

In this study, executive function and working memory improved significantly for CI users, as in some other studies [31,40,66]. HL is known to negatively impact working memory and executive function [67,68,69]. Speech recognition and spoken language comprehension rely on the cognitive processes of attention, learning, memory, and inhibition to support the encoding, processing, storage, and retrieval of linguistic speech information [70,71]. Degraded auditory input must be held longer in working memory, and the process of mapping the acoustic speech signal to lexical representations in long-term memory is impeded, increasing listening effort [72,73]. The information degradation hypothesis postulates that greater resources are employed to store and process this poor-quality information, decreasing performance in other cognitive domains [74]. Although the integrity of the electrical signal received by CI users is vastly degraded relative to the original spoken input [75], the significant improvement in executive function and working memory seen in the CI group in this study and others suggests that improved auditory input decreases cognitive load, facilitating improved function in other cognitive domains.

The current CI–cognition literature focuses on improvements in cognitive function after implantation. However, as cognitive decline is part of the normal process of cognitive aging [76,77], which is accelerated in the presence of HL [3,6,78], an absence of decline in the CI group is also a significant outcome. While the AIBL group either declined in psychomotor function and visual attention at the rate expected due to normal cognitive aging (Table 5; 1.1–1.5% and 2.2–3.9% declines at 18- and 36-month follow-up [74]) or remained stable, the CI group did not decline at all, despite significant HL which declined faster over time. Given the common and often high intra-individual variability in cognitive performance across neuropsychological tests and cognitive domains in normal adults [76,79,80] and the small follow-up sample sizes in this study, these results for CI users are notable.

Surprisingly, to date, no CI study has reported measuring objective device use (treatment compliance). In this study, dose–response effects of device use on cognitive performance were investigated using objective (data logging) data. Participants in this study who used their devices more (≥14 h per day) showed significant and greater magnitudes of improvement on all cognitive subtests except visual learning by 18 months, while those who used their devices less did not demonstrate any significant improvements until the 36-month follow-up. This suggests that greater exposure to auditory stimulation supports a faster neuroplastic response, improving cognitive function more quickly. If confirmed with larger samples, this information could be helpful in advising new CI users on maximising their cognitive benefit from CIs.

As reported previously [38,39,58,59], the CI users with the poorest cognitive baseline performance in this study showed greater magnitudes of improvement in cognitive performance. However, these other studies did not include comparative groups and did not evaluate performance in a manner that allowed regression to the mean to be separated from a real effect of device use. In the current study, significant mean cognitive improvements were consistent across the CI group, with both the AIBL and CI participants who performed more poorly at baseline showing only regression to the mean in subsequent follow-ups, rather than a true effect of poorer performers deriving more cognitive benefit from CIs. As noted in a recent systematic review, regression to the mean is likely the case for at least some other studies [30].

Despite the lengthy timeframe for cognitive decline/dementia and evidence that activation of the auditory cortex continues for at least 3 years after cochlear implantation for speech stimuli [81], only four studies of cognitive performance in CI users to date have extended beyond 2 years ([36,59,82,83], with ongoing follow-up results for two studies published multiple times through to 2024 [82,83]). Studies with short follow-up periods have reported lower magnitudes of improvement [84], but these null results may have been reported due to inadequate follow-up. The current study found no significant overall group improvements until the 36-month follow-up, illustrating the importance of longer-term follow-up. Conversely, two reports from the same study of CI users (n = 75 across mean 4.5-year follow-up [85] and n = 25 over 4-year follow-up [15]) reported significant improvements in cognitive performance 12 months postoperatively, followed by a decline in performance thereafter. In the current study, CI users demonstrated an ongoing trend of improvement in working memory and executive function through to 4.5 years post-implantation. The longer-term cognitive effects of CI use remain unclear and need to be clarified with studies that include longer follow-up periods.

### 4.1. Strengths and Limitations

The strengths of this study include longer follow-up than all but two studies to date [83,85]; a non-intervention comparative group representative of the general population of community-living older adults; the visual-only presentation of a highly sensitive cognitive assessment battery with remote, automated scoring of results to avoid bias/error; the assessment of other dementia risk factors; and the objective measurement of device use, speech perception benefits, and hearing. An additional strength is the examination of outcomes across multiple cognitive domains rather than global assessment, contributing insights into which cognitive domains are most impacted by CI use [86]. Dementia outcomes as well as cognitive performance outcomes are reported, as these are also outcomes of primary interest. With longer follow-up, it may be possible to quantify the effects of CI use on dementia risk.

This study’s limitations include small sample sizes at follow-up, despite statistical power to detect the reported effects. This limitation will be addressed through further recruitment and follow-up. There is also likely bias due to self-selection of participants. Although both groups chose to participate in research studies, many AIBL participants with HL chose not to receive hearing intervention. The CI group, who chose to receive a surgical intervention, could have been significantly more motivated to manage their health conditions. However, the CI group had severe–profound HL and were thus significantly disabled, while 47% of the AIBL group had only average mild HL and the remainder had normal hearing. A further limitation is that although identified differences in participant characteristics at baseline were controlled using fixed effects, differences between groups in an observational study cannot be assumed to be random, and it was not possible to control for unidentified characteristics along the cognitive trajectory. However, the AIBL group had fewer dementia risk factors and significantly better cognitive performance at baseline. The CI group was thus likely at higher risk of cognitive decline than the AIBL group, making it plausible that the direction of bias for the AIBL group was toward that of less cognitive decline than the CI group.

### 4.2. Future Directions

Well-designed, longer-term, large-scale observational studies to further investigate the effects of cochlear implant use on cognitive decline and dementia risk are needed, given that long, randomised clinical trials are ethically impractical with older adults with severe–profound HL due to their significant hearing disability [30,31]. The inclusion of middle-aged adults in such studies, given that HL is now recognized as a mid-life risk factor for dementia [55], would be desirable. Investigations of the effects of CI use could also be informed by concurrent observation of neuroplasticity and biomarkers of cognitive decline. With larger sample sizes and longer follow-up, the stratification of cognitive outcomes according to dementia subtypes may be possible. This would facilitate progress in understanding the effects of CI use on cognitive decline and dementia risk and whether the association between HL and cognitive decline is causal.

### 4.3. Conclusions

Untreated HL has enormous detrimental effects on quality of life for those affected and their families [87], as well as significant economic costs due to lost income and productivity [1]. The results of this study suggest that cochlear implantation may increase cognitive resilience/function, providing proof-of-concept evidence of the effects of hearing intervention with CIs on cognitive function. Cochlear implantation could help to delay the onset of dementia, extending quality of life, promoting healthy aging, and facilitating significant economic benefits. Ongoing screening of auditory status and function in older adults who use hearing aids but may be candidates for CIs is important, as this will enable proactive and effective hearing intervention to support continued social engagement, maintenance of function, and aging well. The promotion of this hearing intervention could be an important strategy for reducing or delaying dementia in adults with severe–profound HL. Given the current very low take-up rates, improved knowledge of CIs (particularly eligibility criteria) in the medical profession and significantly increased referrals would be required to implement this intervention as a successful public health strategy.

## Figures and Tables

**Table 1:** Demographic and audiometric characteristics of CI and AIBL participants at baseline, 18-, 36-, and 54-month follow-up.

	CI Participants				AIBL Participants			CI vs. AIBL (*p*-Value)		
	Baseline	18 Months	36 Months	54 Months	Baseline	18 Months	36 Months			
Age (years)										
n	101	54	37	25	100	47	17			
Mean	73.18	74.97	75.01	76.07	74.39	75.21	77.31	0.118	0.812	0.064
Median	72.7	74.3	74.4	75.7	74.5	74.9	77.4			
S.D.	6.6	6.2	5.0	4.0	4.0	3.8	3.6			
Min	61.3	63.3	64.8	69.0	67.0	68.4	71.4			
Max	90.1	91.7	84.8	83.0	84.8	83.0	84.0			
BPTA										
n	96	45	18	15	100	36	16			
Mean	76.93	81.75	75.97	75.17	20.96	21.46	22.66	**0.000**	**0.000**	**0.000**
Median	77.5	82.5	77.5	81.2	21.2	20.6	21.9			
SD	18.8	20.6	21.8	19.4	8.4	8.5	7.8			
Min	27.5	42.5	47.5	48.8	3.8	6.2	8.8			
Max	118.8	130	116.2	103.8	43.8	40	33.8			
WPTA										
n	96	0	0	0	100	36	16			
Mean	98.78				25.65	25.97	27.03	**0.000**		
Median	96.2				25	25.6	26.9			
SD	15.5				11.2	8.7	8.8			
Min	63.8				8.8	13.8	11.2			
HUI-3 Hearing disability										
n	97	53	36	20						
Mean	0.44	0.62	0.58	0.67						
Median	0.3	0.7	0.7	0.7						
SD	0.2	0.2	0.2	0.1						
Min	0	0	0	0.3						
Max	0.9	1	0.7	0.7						
Normal hearing										
n	96	45	18	15	100	36	16			
No. (%)	0 (0)	0 (0)	0 (0)	0 (0)	47 (47)	18 (50)	8 (50)	**0.000**	**0.000**	**0.002**
Female										
n	101	54	37	25	100	47	17			
No. (%)	42 (41.6)	22 (40.7)	19 (51.4)	10 (40.0)	55 (55.0)	27 (57.4)	10 (58.8)	0.057	0.096	0.619
Education > 15 years										
n	98	54	37	25	100	47	17			
No. (%)	48 (49.0)	29 (53.7)	17 (45.9)	14 (56.0)	72 (72.0)	27 (57.4)	10 (58.8)	**0.001**	0.709	0.392
Cardiovascular condition										
n	97	53	36	19	95	33	12			
No. (%)	73 (75.3)	39 (73.6)	26 (72.2)	15 (78.9)	44 (46.3)	17 (51.5)	7 (58.3)	**0.00**	**0.044**	0.417
Ever smoker										
n	97	15	13	19	47	26	9			
No. (%)	37 (38.1)	5 (33.3)	1 (7.7)	5 (26.3)	17 (36.2)	10 (38.5)	1 (11.1)	0.820	0.750	0.804
1 apolipoprotein E (APOE) ε4 allele										
n	59	46	34	25	99	46	17			
No. (%)	17 (28.8)	11 (23.9)	8 (23.5)	6 (24)	28 (28.3)	16 (34.8)	4 (23.5)	0.944	0.257	1.00
2 apolipoprotein E (APOE) ε4 alleles										
n	59	46	34	25	99	46	17			
No. (%)	3 (5.1)	2 (4.3)	1 (2.9)	0 (0)	1 (1.0)	1 (2.2)	0 (0)	0.187	0.562	0.325
Diabetes										
n	97	53	36	20	96	32	11			
No. (%)	14 (14.4)	9 (17)	4 (11.1)	3 (15)	5 (5.2)	2 (6.2)	2 (18.2)	**0.031**	0.117	0.603
Falls										
n	97	53	36	20	96	33	11			
No. (%)	14 (14.4)	9 (17.0)	4 (11.1)	1 (5.0)	7 (7.3)	3 (9.1)	3 (27.3)	0.112	0.281	0.303
Living arrangements										
Own or rented home with spouse/others										
n	98	54	37	25	97	46	16			
No. (%)	69 (70.4)	37 (68.5)	25 (67.6)	20 (80)	73 (75.3)	36 (78.3)	12 (75)	0.449	0.274	0.590
Own or rented home alone										
n	98	54	37	25	97	46	16			
No. (%)	21 (21.4)	13 (24.1)	9 (24.3)	5 (20)	24 (24.7)	10 (21.7)	4 (25)	0.585	0.784	0.960
Residential aged care										
n	98	54	37	25	97	46	16			
No. (%)	1 (1)	0 (0)	0 (0)	0 (0)	0 (0)	0 (0)	0 (0)	0.320		
Home of relative										
n	98	54	37	25	97	46	16			
No. (%)	2 (2)	1 (1.9)	1 (2.7)	0 (0)	0 (0)	0 (0)	0 (0)	0.158	0.322	0.324
Other (retirement village, motor home)										
n	98	54	37	25	97	46	16			
No. (%)	5 (5.1)	3 (5.6)	2 (5.4)	0 (0)	0 (0)	0 (0)	0 (0)	**0.025**	0.083	0.160
Living alone										
n	98	54	37	25	97	46	16			
No. (%)	21 (21.4)	13 (24.1)	9 (24.3)	5 (20.0)	24 (24.7)	10 (21.7)	4 (25.0)	0.585	0.784	0.960
Anxiety										
n	97	53	36	20	95	33	11			
No. (%)	18 (18.6)	4 (7.5)	3 (8.3)	1 (5.0)	7 (7.4)	3 (9.1)	1 (9.1)	**0.021**	0.806	0.942
Socially isolated										
n	78	53	36	20						
No. (%)	0 (0)	1 (1.9)	1 (2.8)	0 (0)						
Depression										
n	97	53	36	20	96	32	11			
No. (%)	13 (13.4)	3 (5.7)	2 (5.6)	0 (0)	7 (7.3)	0 (0)	2 (18.2)	0.165	0.083	0.343
Retired										
n	98	52	35	19	96	33	12			
No. (%)	77 (78.6)	47 (90.4)	33 (94.3)	16 (84.2)	85 (88.5)	31 (93.9)	(100.0)	0.061	0.549	0.160

Notes: As some CI participants had no measurable hearing at baseline, the number of participants with audiometric data is lower than the total sample size. Participants were implanted in ear with worse hearing. Post-operative hearing was not tested in the implanted ear, as it is unclear whether electrical and acoustic hearing thresholds are equivalent. Bold denotes significant differences (*p* < 0.05; significant at the 5% confidence level). Abbreviations: SD, standard deviation. BPTA: better ear pure tone average, dB hearing level. WPTA: worse ear pure tone average, dB hearing level. PTA ≤ than 20 dB hearing level.

**Table 2:** Cochlear implant use and speech perception scores at baseline, 18-, 36-, and 54-month follow-up.

					*p*-Values		
	Baseline	18 mths	36 mths	54 mths	Baseline vs. 18 mths	18 vs. 36 mths	36 vs. 54 mths
CI usage (hrs/day)							
n		54	36	21			
Mean		12.1	12.8	12.4		**0.045**	0.554
Median		12.8	13.5	13.1			
S.D.		2.9	2.9	3.5			
Min		3.3	1.5	0			
Max		17	19.8	17			
CI usage (% of a 14 h day)							
>90%		51.9	72.2	61.9			
60–90%		37	19.4	33.3			
30–60%		9.3	5.6	0			
<30%		1.9	2.8	4.8			
CVC Words (% correct)							
n	95	51	32	23			
Mean	42.83	70.71	73.75	78.13	**0.000**	0.306	0.264
Median	40	74	78	80			
S.D.	26.2	21.2	16.2	14.8			
Min	0	22	34	24			
Max	94	98	94	96			
CVC Phonemes (% correct)							
n	95	51	32	23			
Mean	65.85	85.41	86.8	91.01	**0.000**	0.540	0.121
Median	67	90	92	92			
S.D.	21.8	13	12.3	8.7			
Min	0.1	51	38	55			
Max	97	99	97	98			
SRT							
n	87	51	32	22			
Mean	10.94	5.05	4.31	3.66	**0.000**	0.111	0.328
Median	9.5	4.6	3.9	3.6			
S.D.	7.2	3.4	2.8	2.3			
Min	−0.9	−0.6	−1.1	−1.1			
Max	21	15	11	9.3			

Bold denotes significant differences (*p* < 0.05; significant at the 5% confidence level). CI use was measured using objective data logging information. Greater CVC Word and Phoneme scores show improved performance in quiet listening conditions. The SRT score measures the signal-to-noise ratio at which 50% of key words are correct in noise; therefore, decreases in SRT scores represent improved performance.

**Table 3:** Baseline scores for CI participants on the Cogstate Groton Maze Learning Task and for CI and AIBL participants on the Cogstate Brief Battery.

	CI	AIBL	CI vs. AIBL (*p*-Value)
	Baseline		
Executive function			
n	101		
Mean	66.74		
Median	56		
S.D.	43.8		
Min	22		
Max	336		
Working memory			
n	101	100	
Mean	2.95	2.93	**0.030**
Median	3.0	2.9	
S.D.	0.1	0.1	
Min	2.8	2.7	
Max	3.2	3.1	
Psychomotor function			
n	101	100	
Mean	2.62	2.54	**0.000**
Median	2.6	2.5	
S.D.	0.1	0.1	
Min	2.4	2.4	
Max	2.9	2.8	
Visual attention			
n	101	100	
Mean	2.78	2.74	**0.000**
Median	2.8	2.7	
S.D.	0.1	0.1	
Min	2.6	2.6	
Max	3.0	2.9	
Visual learning			
n	101	100	
Mean	0.94	1.0	**0.000**
Median	0.9	1.0	
S.D.	0.1	0.1	
Min	0.6	0.7	
Max	1.2	1.2	
MMSE			
n	99	97	
Mean	27.98	28.65	**0.017**
Median	28	29	
S.D.	2.4	1.4	
Min	11	24	
Max	30	30	

Note: Bolding denotes significant *p*-value after Holm–Bonferroni correction.

**Table 4:** Cognition trajectories on the Cogstate Groton Maze Learning Task and the Cogstate Brief Battery for CI participants from baseline to 54-month follow-up.

CI Group Cognition Trajectories					
	Executive Function	Working Memory	Psychomotor Function	Visual Attention	Visual Learning
Intercept	56.381	2.962	2.626	2.784	0.932
p	0.000	0.000	0.000	0.000	0.000
18 mths	−9.825	−0.016	0.030	0.007	0.000
p	0.064	0.129	0.049	0.436	0.993
36 mths	−14.673	−0.026	0.009	−0.009	−0.015
p	**0.006**	0.049	0.596	0.391	0.408
54 mths	−17.579	−0.050	0.018	−0.008	0.022
p	**0.003**	**0.002**	0.373	0.487	0.359
Age	2.049	0.004	0.004	0.003	−0.002
p	0.003	0.005	0.007	0.007	0.181
Female	11.278	−0.024	−0.028	−0.021	0.025
p	0.106	0.196	0.126	0.127	0.192
Higher Ed	−2.560	−0.014	−0.028	−0.010	0.006
p	0.700	0.404	0.113	0.468	0.758
					
R^2^	0.14	0.11	0.12	0.10	0.04
Mean Dep	61.89	2.94	2.63	2.78	0.94
SD Dep	36.99	0.09	0.10	0.07	0.10

N = 54 at 18 months, 37 at 36 months, and 25 at 54 months. Higher Ed: More than 12 years of education. Bolding denotes significant *p*-value after Holm–Bonferroni correction. Abbreviations: Mean dep, mean, dependent variable; SD dep, standard deviation, dependent variable. For Age 70, female, and Higher Ed, *p* values are reported but are not evaluated for significance. These variables have been treated as controls, not variables of interest.

**Table 5:** Cognition trajectories on the Cogstate Brief Battery for AIBL participants (whole group and only those with HL) from baseline to 36-month follow-up.

AIBL Group Cognition Trajectories—Whole Group					
	Executive Function	Working Memory	Psychomotor Function	Visual Attention	Visual Learning
Intercept		2.911	2.535	2.745	1.030
p		0.000	0.000	0.000	0.000
18 mths		−0.008	0.039	0.032	0.013
p		0.460	**0.009**	**0.001**	0.364
36 mths		0.002	0.099	0.061	0.014
p		0.928	**0.000**	**0.015**	0.524
Age		0.007	0.004	0.003	−0.006
p		0.000	0.032	0.070	0.006
Female		0.000	0.017	−0.016	0.017
p		0.982	0.294	0.187	0.378
Higher Ed		−0.017	−0.030	−0.014	−0.012
p		0.302	0.086	0.255	0.565
					
R^2^		0.12	0.22	0.17	0.07
Mean Dep		2.93	2.57	2.76	1.01
SD Dep		0.08	0.10	0.07	0.10
**AIBL Group Cognition Trajectories—Participants with HL only**					
Intercept		2.929	2.531	2.750	1.038
p		0.000	0.000	0.000	0.000
18 mths		−0.004	0.047	0.026	0.005
p		0.762	**0.015**	0.036	0.768
36 mths		−0.011	0.089	0.033	-0.018
p		0.669	**0.001**	0.305	0.508
Age-70		0.007	0.008	0.005	-0.008
p		0.004	0.004	0.020	0.010
Female		−0.033	−0.003	−0.030	0.018
p		0.077	0.908	0.072	0.473
Higher Ed		−0.035	−0.050	−0.028	0.008
p		0.059	0.050	0.113	0.786
					
R^2^		0.22	0.31	0.22	0.12
Mean Dep		2.93	2.57	2.76	1.01
SD Dep		0.08	0.10	0.07	0.10

Whole group—n = 47 at 18 months and 17 at 36 months. HL-only group—n = 30 at 18 months and 11 at 36 months. Higher Ed: More than 12 years of education. Bolding denotes significant *p*-value after Holm–Bonferroni correction. Abbreviations: Mean dep—mean, dependent variable; SD dep—standard deviation, dependent variable. For Age 70, female, and Higher Ed, *p* values are reported but are not evaluated for significance. These variables have been treated as controls, not variables of interest.

**Table 6:** CI group cognition trajectories relative to amount of device use.

	Executive Function	Working Memory	Psychomotor Function	Visual Attention	Visual Learning
Intercept	56.372	2.961	2.626	2.783	0.932
p	0.000	0.000	0.000	0.000	0.000
18 mth	−5.138	−0.004	0.047	0.017	−0.006
p	0.406	0.765	0.006	0.130	0.716
36 mth	−17.979	−0.025	0.017	−0.013	−0.015
p	0.003	0.137	0.373	0.267	0.526
54 mth	−14.082	−0.049	0.035	−0.001	−0.008
p	0.032	0.02	0.181	0.921	0.785
High CI Use, 18 mth	−19.56	−0.052	−0.07	−0.041	0.026
p	**0.016**	**0.019**	**0.013**	**0.000**	0.354
High CI Use, 36 mth	11.42	−0.001	−0.013	0.022	0.001
p	0.184	0.979	0.796	0.435	0.978
High CI Use, 54 mth	−6.758	0.028	−0.029	−0.002	0.066
Age-70	2.061	0.004	0.004	0.003	−0.002
p	0.003	0.002	0.005	0.003	0.181
Female	11.184	−0.022	−0.03	−0.021	0.022
p	0.116	0.197	0.097	0.127	0.263
Higher Ed	−2.539	−0.014	−0.028	−0.008	0.009
					
p	0.705	0.384	0.101	0.531	0.63
R2	0.16	0.12	0.15	0.12	0.04
Mean dep	62.18	2.95	2.63	2.78	0.94
SD dep	37.31	0.09	0.10	0.07	0.10

N = 54 at 18 months, 37 at 36 months, and 25 at 54 months. Higher Ed: More than 12 years of education. Bolding denotes significant *p*-value after Holm–Bonferroni correction. Abbreviations: Mean dep, mean, dependent variable; SD dep, standard deviation, dependent variable. For Age 70, female, and Higher Ed, *p* values are reported but are not evaluated for significance. These variables have been treated as controls, not variables of interest.

**Table 7:** Results of multiple regression analyses of comparative cognitive trajectories on the Cogstate Brief Battery for the CI vs. AIBL groups at 18- and 36-month follow-up.

CI vs. AIBL Trajectories				
	Working Memory	Psychomotor Function	Visual Attention	Visual Learning
18 mths	0.006	0.042	0.039	0.000
p	0.550	0.003	0.000	0.986
36 mths	0.024	0.104	0.067	−0.025
p	0.284	0.000	0.004	0.266
18 mths CI	−0.014	−0.018	−0.028	−0.006
p	0.341	0.381	**0.021**	0.738
36 mths CI	−0.027	−0.087	−0.060	0.003
p	0.275	**0.001**	**0.017**	0.927
				
**CI vs. AIBL trajectories (AIBL participants with HL only)**				
		
18 mths	−0.001	0.047	0.032	−0.006
p	0.950	0.008	0.004	0.683
36 mths	0.011	0.105	0.054	−0.016
p	0.670	0.000	0.071	0.438
18 mths CI	−0.007	−0.023	−0.021	−0.001
p	0.633	0.317	0.124	0.978
36 mths CI	−0.014	−0.088	−0.047	−0.007
p	0.616	**0.002**	0.134	0.785

CI group: n = 54 at 18 months, 37 at 36 months. AIBL (whole) group: n = 47 at 18 months, 17 at 36 months. HL-only group: n = 30 at 18 months and 11 at 36 months. Negative scores indicate improvement, except for visual learning (OCL), which is scored in reverse. Bolding denotes significant *p*-value after Holm–Bonferroni correction.

**Table 8:** Results of sensitivity analyses for differences in education between the CI and AIBL groups.

CI vs. AIBL (Whole Group) Trajectories				
	Working Memory	Psychomotor Function	Visual Attention	Visual Learning
18 mths	0.003	0.070	0.050	−0.001
p	0.864	0.000	0.000	0.941
36 mths	0.067	0.181	0.125	−0.009
p	0.096	0.000	0.000	0.822
18 mths CI	0.004	−0.037	−0.040	−0.019
p	0.876	0.255	0.010	0.420
36 mths CI	−0.055	−0.172	−0.118	−0.019
p	0.206	0.000	0.001	0.682
Higher Ed 18 mths AIBL	0.006	−0.048	−0.019	0.002
p	0.764	0.073	0.293	0.939
Higher Ed 36 mths AIBL	−0.074	−0.130	−0.099	−0.028
p	0.109	**0.000**	0.020	0.571
Higher Ed 18 mths CI	−0.026	−0.018	0.002	0.025
p	0.186	0.576	0.912	0.322
Higher Ed 36 mths CI	−0.031	0.021	0.000	0.010
p	0.139	0.450	0.995	0.750
				
**CI vs. AIBL (HL Only) Trajectories**				
18 mths	0.016	0.067	0.048	−0.016
p	0.214	0.014	0.005	0.334
36 mths	0.068	0.181	0.136	−0.032
p	0.172	0.000	0.011	0.458
18 mths CI	−0.010	−0.034	−0.038	−0.004
p	0.636	0.380	0.048	0.854
36 mths CI	−0.056	−0.173	−0.129	0.004
p	0.285	0.000	0.020	0.937
Higher Ed 18 mths AIBL	−0.027	−0.032	−0.025	0.016
p	0.178	0.366	0.265	0.547
Higher Ed 36 mths AIBL	−0.091	−0.122	−0.132	0.026
p	0.108	**0.005**	0.024	0.590
Higher Ed 18 mths CI	−0.026	−0.018	0.002	0.025
p	0.187	0.576	0.912	0.322
Higher Ed 36 mths CI	−0.031	0.021	0.000	0.010
p	0.140	0.450	0.995	0.750

CI group: n = 54 at 18 months, 37 at 36 months. AIBL (whole) group: n = 47 at 18 months, 17 at 36 months. HL-only group: n = 30 at 18 months and 11 at 36 months. Higher Ed: More than 12 years of education. Controls in this analysis were age, sex, and education: each interacted with follow-up period indicators. Negative scores indicate improvement, except for visual learning (OCL), which is scored in reverse. Bolding denotes significant *p*-value after Holm–Bonferroni correction.

**Table 9:** CI and AIBL cognitive performance trajectories relative to baseline performance.

Timepoint	Working Memory	Psychomotor Function	Visual Attention	Visual Learning
				
18 mths	18.114	9.507	16.643	5.132
p	0.001	0.095	0.011	0.033
18 mths CI	−2.209	7.366	−2.211	0.275
p	0.746	0.335	0.802	0.896
36 mths	22.366	8.917	9.087	1.294
p	0.002	0.537	0.783	0.662
36 mths CI	−15.759	−3.737	1.402	−2.075
p	0.070	0.802	0.966	0.381
Baseline: 18 mths	−0.442	−0.490	−0.444	−0.385
p	**0.006**	0.024	**0.004**	0.026
Baseline: 36 mths	−0.679	−0.462	−0.452	−0.549
p	**0.000**	0.364	0.558	**0.008**
Baseline: 18 mths CI	0.060	−0.245	0.045	−0.059
p	0.757	0.355	0.821	0.783
Baseline: 36 mths CI	0.430	0.105	−0.048	0.186
p	0.077	0.843	0.950	0.425
Age-70 18 mths	0.022	0.046	0.035	−0.022
p	0.326	0.055	0.019	0.261
Age-70 36 mths	0.010	0.021	0.045	0.049
p	0.697	0.463	0.106	0.052
Female 18 mths	−0.303	−0.119	−0.270	0.114
p	0.082	0.537	0.134	0.535
Female 36 mths	−0.561	0.085	−0.338	0.059
p	0.010	0.748	0.126	0.823
Higher Ed 18 mths	−0.173	−0.331	−0.068	0.085
p	0.317	0.091	0.694	0.637
Higher Ed 36 mths	−0.398	−0.247	−0.370	0.196
p	0.055	0.361	0.190	0.417
				

CI group: n = 54 at 18 months, 37 at 36 months. AIBL group: n = 47 at 18 months, 17 at 36 months. Higher Ed: More than 12 years of education. Controls in this analysis were age, sex, and education: each interacted with follow-up period indicators. Negative scores indicate improvement, except for visual learning (OCL), which is scored in reverse. Bolding denotes significant *p*-value after Holm–Bonferroni correction.

## Data Availability

The datasets used in this study are not available as the ethical consent provided by participants does not permit use of the data beyond the current study. The publication of these data does not compromise the anonymity of the participants or breach local data protection laws.

## Appendix A. Derivations of Trajectory Equations

The trajectory Equation (1) is derived from the simple representation

(A1) $$Y_{i,t}=\mu_{t}+\psi^{\prime }X_{i}+U_{i,t},\;t=0,1,2,$$

where $X_{i}$ is the vector of controls (age, sex, and education) and t=0,1,2 refers to baseline, 18-month, and 36-month time points, respectively. The parameters $\mu_{0},\mu_{1},\mu_{2}$ denote mean cognition at time points t=0,1,2 for $X_{i}=0$ (i.e., for 70-year-old males without higher education). The disturbances $U_{i,t}$ have a mean of zero and $\mathrm{var}\left(U_{i,t}\right)=\sigma_{t}^{2}$ for t=0,1,2, and $\mathrm{cov}\left(U_{i,t},U_{i,0}\right)=\rho_{t}/\left(\sigma_{t}\sigma_{0}\right)$ for t=1,2, allowing for intra-individual correlations amongst the disturbances, as well as varying variances and covariances across time points. Equation (A1) can be rearranged to give

(A2) $$\begin{array}{ll} Y_{i,t} & =\mu_{0}+\left(\mu_{1}-\mu_{0}\right)D_{1,t}+\left(\mu_{2}-\mu_{0}\right)D_{2,t}+\psi \prime X_{i}+U_{i,t}\\ & =\mu_{0}+\delta_{1}D_{1,t}+\delta_{2}D_{2,t}+\psi^{\prime }X_{i}+U_{i,t} \end{array}$$

where $D_{1,t}$ and $D_{2,t}$ are indicators for the 18-month and 36-month time periods. This is trajectory Equation (1), which can be estimated separately for each of the CI and AIBL groups.

For comparing the CI and AIBL groups, separate equations of the form (A2) can be combined as

(A3) $$\begin{array}{ll} Y_{i,t} & =\mu_{0}+\delta_{1}D_{1,t}+\delta_{2}D_{2,t}\\ & \;\;+\lambda_{0}{\mathrm{CI}}_{i}+\lambda_{1}\left(D_{1,t}\times {\mathrm{CI}}_{i}\right)+\lambda_{2}\left(D_{2,t}\times {\mathrm{CI}}_{i}\right)\\ & \;\;+\psi^{\prime }X_{i}+U_{i,t}. \end{array}$$

Equation (A3) results from combining the time invariant terms $\mu_{0}+\lambda_{0}{\mathrm{CI}}_{i}+\psi \prime X_{i}$ into the generic fixed effects $\alpha_{i}$, noting that $\alpha_{i}$ also controls for other observed or unobserved baseline characteristics that may differ between the two groups.

To derive the equation relating cognition trajectories to baseline cognition, first note that for t=1,2, the covariance structure of $U_{i,t}$ permits the representation

(A4) $$\begin{array}{ll} \frac{U_{i,t}}{\sigma_{t}} & =\rho_{t}\frac{U_{i,0}}{\sigma_{0}}+V_{i,t}\\ & =\rho_{t}\left(\frac{Y_{i,0}}{\sigma_{0}}-\frac{\mu_{0}}{\sigma_{0}}-\frac{\lambda_{0}}{\sigma_{0}}{\mathrm{CI}}_{i}-\frac{\psi \prime }{\sigma_{0}}X_{i}\right)+V_{i,t} \end{array}$$

where $U_{i,0}$ and $V_{i,t}$ are uncorrelated by construction. For t=1, (A3) and (A4) combine to give

(A5) $$\begin{array}{ll} \frac{Y_{i,1}}{\sigma_{1}}-\frac{Y_{i,0}}{\sigma_{0}} & =\left(\rho_{1}-1\right)\frac{Y_{i,0}}{\sigma_{0}}+\left(\frac{\mu_{0}}{\sigma_{1}}+\frac{\delta_{1}}{\sigma_{1}}\right)+\left(\frac{\lambda_{0}}{\sigma_{1}}+\frac{\lambda_{1}}{\sigma_{1}}\right){\mathrm{CI}}_{i}+\frac{\psi \prime }{\sigma_{1}}X_{i}\\ & \;\;-\rho_{1}\left(\frac{\mu_{0}}{\sigma_{0}}+\frac{\lambda_{0}}{\sigma_{0}}{\mathrm{CI}}_{i}+\frac{\psi \prime }{\sigma_{0}}X_{i}\right)+V_{i,1}\\ & =\left(\rho_{1}-1\right)\frac{Y_{i,0}}{\sigma_{0}}+\delta_{1}^{*}+\lambda_{1}^{*}{\mathrm{CI}}_{i}+\psi_{1}^{*\prime }X_{i}+V_{i,1}, \end{array}$$

and similarly, for t=2:

(A6) $$\begin{array}{ll} \frac{Y_{i,2}}{\sigma_{2}}-\frac{Y_{i,0}}{\sigma_{0}} & =\left(\rho_{2}-1\right)\frac{Y_{i,0}}{\sigma_{2}}+\delta_{2}^{*}+\lambda_{2}^{*}{\mathrm{CI}}_{i}+\psi_{2}^{*\prime }X_{i}+V_{i,2}, \end{array}$$

where for t=1,2:

$$\begin{array}{l} \delta_{t}^{*}=\frac{\mu_{0}+\delta_{t}}{\sigma_{t}}-\rho_{t}\frac{\mu_{0}}{\sigma_{0}},\;\lambda_{t}^{*}=\frac{\lambda_{0}+\lambda_{t}}{\sigma_{t}}-\rho_{t}\frac{\lambda_{0}}{\sigma_{0}},\;\psi_{t}^{*}=\psi \left(\frac{1}{\sigma_{t}}-\rho_{t}\frac{1}{\sigma_{0}}\right). \end{array}$$

Together, Equations (A5) and (A6) demonstrate the “regression to the mean” effect that standardised cognitive changes since baseline at each follow-up will be negatively correlated with baseline cognition. This is essentially a statistical artifact whose strength is determined by the intra-individual correlations $\rho_{1}$ and $\rho_{2}$, and not evidence that the CI treatment effects $\lambda_{1}$ and $\lambda_{2}$ are negatively correlated with baseline cognition. A testing equation can, however, be derived for the possibility that $\lambda_{1}$ and $\lambda_{2}$ are negatively correlated with standardised baseline cognition, that is:

$$\lambda_{t}=\lambda_{t}^{\left(0\right)}+\lambda_{t}^{\left(1\right)}\frac{Y_{i,0}}{\sigma_{0}}$$

in which $\lambda_{t}^{\left(1\right)}$ may be expected to be negative. Substituting this into the expression for $\lambda_{t}^{*}$ and hence into (A5) and (A6) leads to the representations

$$\begin{array}{ll} \frac{Y_{i,1}}{\sigma_{1}}-\frac{Y_{i,0}}{\sigma_{0}} & =\left(\rho_{1}-1\right)\frac{Y_{i,0}}{\sigma_{0}}+\phi_{1}\left({\mathrm{CI}}_{i}\times \frac{Y_{i,0}}{\sigma_{0}}\right)+\delta_{1}^{*}+\lambda_{1}^{*}{\mathrm{CI}}_{i}+\psi_{1}^{*\prime }X_{i}+V_{i,1}\\ \frac{Y_{i,2}}{\sigma_{2}}-\frac{Y_{i,0}}{\sigma_{0}} & =\left(\rho_{2}-1\right)\frac{Y_{i,0}}{\sigma_{0}}+\phi_{2}\left({\mathrm{CI}}_{i}\times \frac{Y_{i,0}}{\sigma_{0}}\right)+\delta_{2}^{*}+\lambda_{2}^{*}{\mathrm{CI}}_{i}+\psi_{2}^{*\prime }X_{i}+V_{i,2} \end{array}$$

where

$$\phi_{t}=\frac{\lambda_{t}^{\left(1\right)}}{\sigma_{t}},\;t=1,2$$

are the parameters that represent any correlation in the CI treatment effect $\lambda_{t}$ with baseline cognition $Y_{i,0}$. Testing Equation (5) consists of these two equations combined into a single equation using the period indicators.

## Appendix B. Analysis of Withdrawals

There were 54 withdrawals in total from the CI group. Such withdrawals need not bias the analysis, but they might introduce bias if the pattern of withdrawals is systematically associated with the outcomes of interest. The following table presents a breakdown of the reasons for withdrawal from the study for the CI group.

**Table A1:** The reasons for withdrawal from the study for the CI group.

Reason	Baseline	18 mth	36 mth	54 mth
Incomplete baseline	12	0	0	0
Unresponsive to follow-up	5	0	0	0
Death	2	2	1	0
Dementia/neurological deterioration	0	0	1	0
Ill health (physical)	5	3	0	0
Poor CI outcomes/non-user	5	1	0	0
Other	4	0	2	2
Inappropriate recruit	2	0	0	0
Closure of test site	5	3	0	0
Total	40	9	4	2

It is plausible that, on average, participants who withdrew for any of the first six reasons may have had poorer cognitive performance. It is implausible that any of these reasons would be consistent with improved cognition. The final three reasons do not have any clear connection with cognitive outcomes, and the numbers are small. It is possible that withdrawals of participants with more risk factors for worse cognitive performance may bias the results towards improved cognition for CI participants who remained in the study. A sensitivity analysis was performed to evaluate this possibility.

Most withdrawals occurred at baseline, with those participants reporting finding questionnaire completion in combination with the cognitive assessments too onerous. If the results of only the 40 participants who withdrew following baseline cognition testing were removed from the analysis and the above conjecture were true (these participants had worse cognitive performance on average), the estimates of baseline cognition would become poorer. Since we have no follow-up observations for these 40 participants, removing them from the analysis would have no effect on the cognitive performance means at 18, 36, or 54 months. The resulting changes from baseline to each follow-up period with this reduced sample would therefore be worse than for the full sample. This analysis can be performed while retaining the relatively small number (*n* = 15) of participants who withdrew later in the study, noting that the conjecture above would imply worse CI effects on cognitive performance for these participants.

This sensitivity analysis is a stern test of any possible bias induced by the withdrawals: the baseline withdrawals whose inclusion may increase any positive effects of CI use on cognitive performance are omitted, while the later withdrawals after follow-ups whose inclusion may decrease the CI effect are retained.

The table below shows the CI cognition trajectories estimated from the sample with all baseline withdrawals omitted. These results can be compared with those in the top panel of Table 4 showing cognition trajectories, and are changed very little by this sensitivity analysis. In fact, counter to the conjectured bias above, there are two additional significant findings of improvements in CI participant cognitive performance: in executive performance (GML) at 18 months and working memory (ONB) at 36 months.

This sensitivity analysis demonstrates clearly that the CI participant results are not being driven by any bias associated with participant withdrawals.

**Table A2:** CI group cognitive trajectories with baseline withdrawals omitted.

	GML	ONB	DET	IDN	OCL
Intercept	60.562	2.966	2.655	2.797	0.924
p	0.000	0.000	0.000	0.000	0.000
18 mth	−9.412	−0.024	−0.001	−0.004	−0.004
p	0.036 *	0.050	0.965	0.695	0.796
36 mth	−15.554	−0.039	−0.021	−0.026	−0.020
p	0.015 *	0.004 *	0.245	0.028	0.331
54 mth	−14.322	−0.055	−0.004	−0.019	0.008
p	0.024 *	0.002 *	0.847	0.134	0.759
Age-70	1.022	0.004	0.005	0.003	0.001
p	0.160	0.036	0.059	0.045	0.678
Female	12.973	−0.023	−0.036	−0.025	0.040
p	0.141	0.324	0.130	0.151	0.103
Higher Ed	−6.009	−0.023	−0.040	−0.018	−0.009
p	0.438	0.300	0.088	0.271	0.690
R2	0.10	0.12	0.13	0.13	0.05
Mean dep	59.00	2.94	2.63	2.78	0.94
SD dep	30.97	0.08	0.10	0.06	0.10

* Significant at 0.05 level after Holm–Bonferroni corrections.
